# Biosafety steps in the manufacturing process of spray-dried plasma: a review with emphasis on the use of ultraviolet irradiation as a redundant biosafety procedure

**DOI:** 10.1186/s40813-020-00155-1

**Published:** 2020-07-16

**Authors:** Elena Blázquez, Carmen Rodríguez, Jesús Ródenas, Joaquim Segalés, Joan Pujols, Javier Polo

**Affiliations:** 1 APC EUROPE, S.L., Avda, Sant Julià 246-258, Pol. Ind. El Congost, E-08403 Granollers, Spain; 2 IRTA, Centre de Recerca en Sanitat Animal (CReSA, IRTA-UAB), Campus de la Universitat Autònoma de Barcelona, 08193 Bellaterra, Barcelona Spain; 3 OIE Collaborating Centre for the Research and Control of Emerging and Re-emerging Swine Diseases in Europe (IRTA-CReSA), Bellaterra, Barcelona Spain; 4 Departament de Sanitat i Anatomia Animals, Universitat Autònoma de Barcelona (UAB), 08193 Bellaterra, Barcelona Spain; 5 UAB, Centre de Recerca en Sanitat Animal (CReSA, IRTA-UAB), Campus de la Universitat Autònoma de Barcelona, 08193 Bellaterra, Barcelona Spain; 6 APC LLC, 2425 SE Oak Tree Court, Ankeny, IA 50021 USA

## Abstract

Spray dried plasma (SDP) is a functional protein source obtained from blood of healthy animals, approved by the veterinary authorities from animals declared to be fit for slaughter for human consumption. Blood of these animals is collected at the slaughterhouse, treated with an anticoagulant, chilled and transported to industrial facilities in which blood is centrifuged to separate the red blood cells from the plasma fraction. Plasma is then concentrated, and spray dried at high temperatures (80 °C throughout its substance) to convert it in a powder. Such method preserves the biological activity of its proteins, mainly albumins and globulins. SDP is mainly used in pig feed diets to significantly improve daily gain, feed intake, production efficiency, and to reduce post-weaning lag caused by the appearance of post-weaning diarrhea. Although SDP is considered a safe product and its manufacturing process consists of several biosafety steps, the security of the SDP is often questioned due to its nature as raw blood by-product, especially when emergent or re-emergent pathogens appear. This review provides an evaluation and validation of the different safety steps present in the manufacturing process of SDP, with special focus on a new redundant pathogen inactivation step, the UV-C irradiation, that may be implemented in the manufacturing process of the SDP. Overall results showed that the manufacturing process of SDP is safe and the UV-C radiation was effective in inactivating a wide range of bacteria and viruses spiked and naturally present in commercially collected liquid animal plasma and it can be implemented as a redundant biosafety step in the manufacturing process of the SDP.

## Background

Spray-dried plasma (SDP) is a functional protein source obtained from blood of healthy animals approved to be sacrificed for human consumption after veterinary inspection. Blood of these animals is collected at the slaughterhouse, treated with an anticoagulant, chilled and transported to industrial facilities where the blood is centrifuged to separate the red blood cells (RBC) from the plasma fraction. Alternatively, the blood may be centrifuged in the abattoir and then the chilled plasma transported to the manufacturing plant. Plasma is subsequently concentrated either by membrane filtration or vacuum evaporation and spray-dried at high temperatures (80 °C throughout its substance) to convert it to powder. This process preserves the biological activity of the proteins, mainly albumin and globulins, with immunoglobulin G (IgG) as the predominant antibody type [1]. The SDP is produced from porcine (SDPP) or bovine (SDBP) blood and is commonly used in human food and animal feed [1, 2].

SDP has been used as a protein source in piglet feed since the late 1980s [2, 3] and is typically used at an inclusion level between 4 and 8% in the feed [4–6]. The use of SDP in feed for weaned pigs significantly improves daily gain, feed intake, production efficiency, and piglet survival [4–6] compared to other specialty protein sources. SDP in feed reduces diarrhea and the post-weaning growth lag associated with weaning stress [7–9].

Although the well documented benefits of SDP on animal health and performance have long been established, its safety may be questioned particularly in scenarios of emerging or re-emerging diseases in animal populations because it is produced from the abattoir collected animal blood. Thus, the objective of the present manuscript is to review the different biosafety steps present in the manufacturing process of SDP with special focus on the development and adaptation of UV-C irradiation of liquid plasma as an additional biosecurity step that has recently been incorporated in the manufacturing process at some facilities. This review provides detailed information to the stakeholders of the swine industry about the biosafety features and standards used by manufacturers of spray-dried plasma that assure the overall safety of SDP in feed for swine.

## Industrial production of spray-dried plasma and its biosafety steps

**Fig. 1:**
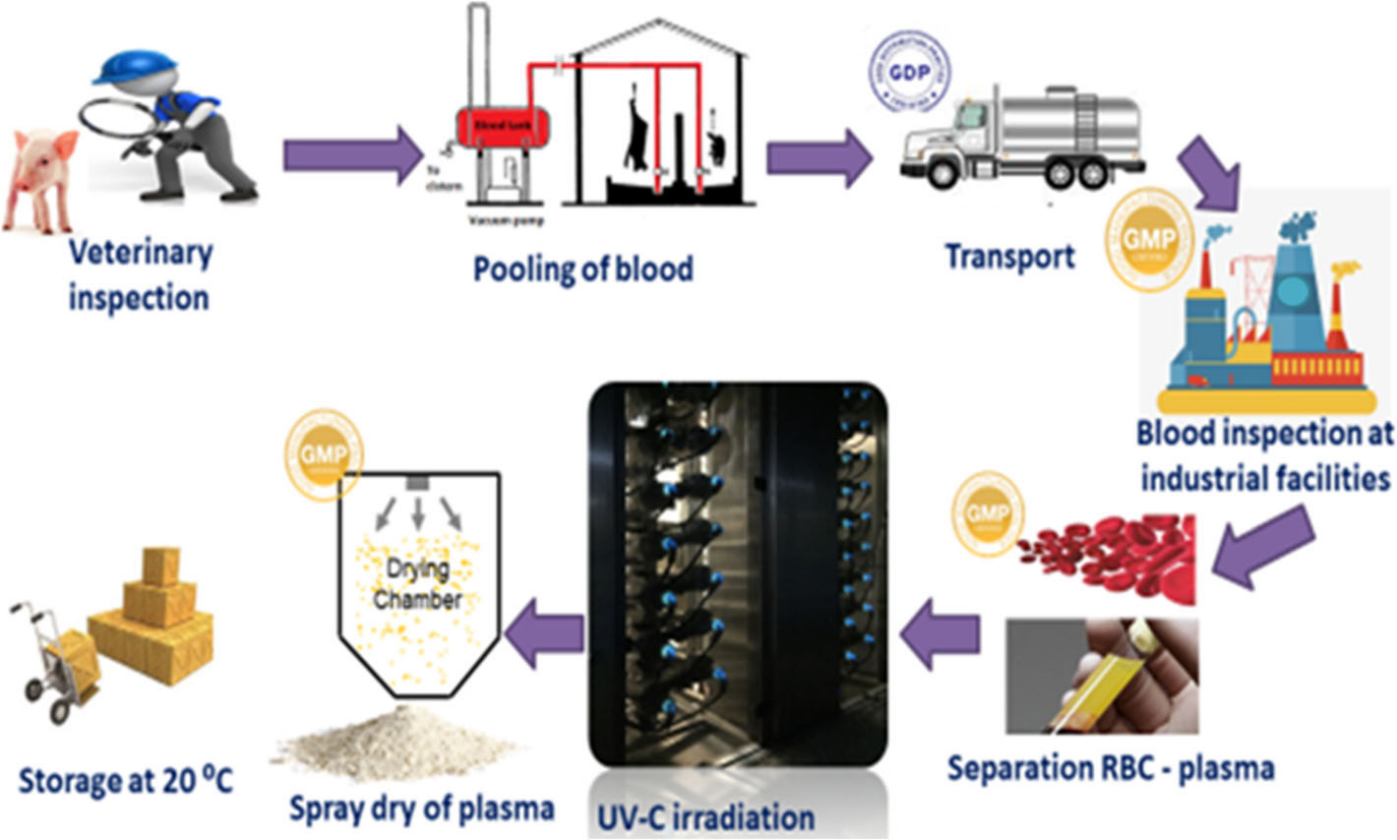
Manufacturing process of spray-dried plasma and its biosafety steps

Commercial production of SDP is done following good manufacturing practices (GMP) using high-quality standards to produce a safe high-quality product. SDP is produced from fresh animal blood as a raw material that requires several safety steps in its production process to eliminate risks for potential biohazards. There are numerous safety features in the industrial manufacturing process of SDP that effectively and collectively reduce biohazard risks to produce a safe final product (Fig. 1). The manufacturing process of SDP has several stages as discussed below.

### Blood collection at the abattoir

The first biosafety step in the production process of SDP starts with the collection of the raw material. Blood from healthy animals, passed as fit for slaughter for human consumption, is collected at abattoirs under inspection by competent authorities. Blood is collected in a stainless-steel pan with anticoagulants added to prevent blood clotting. Sodium citrate [10] or sodium tripolyphosphate [11] are anticoagulants typically used for SDP production. The collection and mixture of the blood from multiple animals contain inherent neutralizing antibodies against numerous habitual pathogens. These inherent neutralizing antibodies may reduce infectivity potential even before further processing steps are done and contribute to the biosafety of the final product [12–14].

To produce SDP, blood is only collected while the carcass is entire, thus minimizing exposure to other tissues. In addition, the blood collection system is separate from the rest of the carcass processing chain. The entire manufacturing process from the time that the blood is collected to the final packaged product is done using a closed system and avoids the possibility of cross contamination with other tissues or from the external environment. In North America, the collection system at some abattoirs delivers the blood to an industrial centrifuge to separate RBC and plasma. After separation, the plasma is concentrated or not concentrated and refrigerated at 4 °C and transported to the processing plant. In Europe, Latin America and other North American abattoirs the collection system delivers the whole blood to stainless steel containers that are kept refrigerated until subsequent transport to the processing plants.

Once the daily blood collection is completed, the entire collection circuit is subjected to a clean in place (CIP) process using food grade approved sanitizing agents. The CIP process ensures proper sanitization of the circuit.

### Transport to the processing plants

Blood or plasma collected at the slaughterhouse is stored in refrigerated containers and transported in isothermal trucks that are sealed after filling. Each daily production lot is identified, and full traceability is retained.

After the transport truck arrives at the processing plant, a subjective inspection of the freshness of the product is done and temperature of the liquid is measured to determine if the product is suitable for processing.

When the truck is emptied, a CIP process is done to assure the cleaning and sanitation of the tank before the next collection is done.

### Processing and spray-drying

At manufacturing plants that receive whole blood, the blood is delivered to a storage tank and passed through a closed system to an industrial centrifuge to separate the plasma from the RBC fraction. The RBC are stored for subsequent production of other products. Before spray drying, the plasma is concentrated with membranes using either nanofiltration or reverse osmosis. Alternatively, concentration by a vacuum evaporator may be used at some plants. After concentration, the plasma is pumped into an industrial spray-dryer, which rapidly dries the concentrated liquid plasma into powder.

Spray-drying involves the atomization of a liquid feed material into a stream of heated air resulting in rapid desiccation. The spray-drying process involves four stages of operation that affect microbial survival and the characteristics of the resulting product: 1) atomization of the liquid source to form droplets into a hot chamber; 2) contact between the spray and the drying medium consisting of very hot air, at a high gas mass to liquid mass flow volume ratio; 3) rapid moisture evaporation resulting in particle formation; and 4) separation of dried particles from the air stream [15–17] following a residence time between 20 and 90 s.

During the drying process, droplets interact with the hot air in the spraying chamber. As moisture is removed the temperature of the dried particle increases to a value similar to the outlet air temperature [18, 19]. Inlet and outlet temperatures have a major influence on the inactivation of microorganisms, but the outlet temperature has the highest impact and is the primary critical control point for the spray-drying process to inactivate microorganisms [20]. The European Animal Protein Association (EAPA) and the North American Spray Dried Blood and Plasma Producers (NASDBPP) have established ≥80 °C for the outlet temperature as a good manufacturing process standard for microbial inactivation during the SDP manufacturing process (www.eapa.biz).

Relatively high drying temperatures, rapid changes in temperature, and rapid dehydration are the phenomena involved in microbial inactivation. Dehydration causes damages in the cells, mainly in the cytoplasmic membrane [21, 22] and also produces damage to DNA, RNA and proteins [23] inactivating many microorganisms [20, 22–27].

Many experiments of deliberated inoculation of pathogens in plasma have demonstrated that spray-drying is a very effective technology to inactivate important pathogens of interest in the swine industry. Pathogen inactivation results from studies using spray drying of plasma inoculated with *Salmonella enterica* [28, 29]*, E.coli* enterotoxigenic strains [30], *Porcine reproductive and respiratory syndrome virus* (PRRSV) [31]*, Pseudorabies virus* (PRV) [31]*, Swine vesicular disease virus* (SVDV) [32], *Porcine epidemic diarrhea virus* (PEDV) [33, 34] or *African swine fever virus* (ASFV) [35] are summarized in Table 1*.* Furthermore, in Table 2 results from other studies are summarized demonstrating the lack of pathogen transmission in pigs provided feed with SDP containing virus genome. There was no transmission of *Porcine circovirus 2* (PCV-2), when naïve pigs were fed diets with SDPP containing genome copies of PCV-2 [36–38, 43]. PCV-2 is known to be one of the most thermal resistant viruses of swine [44, 45]. Also, SDPP containing genome of PEDV has been reported to not be infective when fed to millions of naïve pigs [40, 41]. A retrospective study of different SDPP samples and sera from pigs provided feed with SDPP collected over time, showed that SDPP containing RNA and antibodies of *Hepatitis E virus* (HEV) did not transmit HEV to pigs [39]. These results demonstrate that detection of virus genome in spray-dried blood products should not be interpreted as an infectious material, only that the virus genome segments are detectable and present in the material [46]. Virus isolation or bioassay techniques are necessary to distinguish if virus genome segments detected in SDP can cause infection.

**Table 1:** Reduction factors obtained for different viruses subjected to the spray-drying treatment

Virus	Nucleic acid	Envelope	Virus size (nm)	Virus inactivation expressed as Log 10 TCID_**50**_	Reference
**PRRSV**	ssRNA	Yes	50–65	4.0 log	[31]
**PEDV**	ssRNA	Yes	90–190	> 5.2 log	[34]
**PEDV**	ssRNA	Yes	90–190	> 3.6 log	[33]
**PRV**	ssDNA	Yes	150–180	5.3 log	[31]
**ASFV**	dsDNA	Yes	80–200	~ 4.0 log	[35]
**SVDV**	ssRNA	No	22–30	6.0 log	[32]

**Table 2:** Studies that demonstrated lack of transmission of different swine viruses when PCR genome copies were present in SDP

Virus	PCR genome copies	SDP inclusion level in feed	Feeding duration	Results	Reference
**PCV-2**	2.47 × 10^5.0^	8%	45 days	Not infective	[36]
**PCV-2**	10^6.7^	4%	42 days	Not infective	[37]
**PCV-2**	7.56 × 10^5.0^	8%	32 days	Not infective	[38]
**HEV**	Positive	8%	28 days	Not infective	[39]
**PEDV**	Positive (Ct: 30.1)	5%	14 days	Not infective	[40]
**PEDV**	Positive	3–8%	7 to 14 days	Not infective	[41]
**PRRSV**	Positive	3–8%	7 to 21 days	Not infective	[42]

### Packaging, storage, traceability and quality control

As SDP is being produced, the powder is blended and stored in a small silo, then directly bagged in new packaging materials ensuring no cross contamination after SDP production. All manufactured lots are submitted to standard physicochemical and microbiological analysis that confirm all commercial lots meet specifications.

### Post drying heat treatment and storage time

The low moisture (< 9%) and very low water activity (a_w_ < 0.6) of SDP significantly reduce pathogen survival, especially for bacteria and enveloped viruses during prolonged periods of storage [47].

As an additional safety feature, most manufacturers package and store porcine SDP (SDPP) at room temperature (> 20 °C) for at least 14 days before release for sale. These storage conditions of SDPP demonstrated to be effective in inactivating certain pathogens susceptible to dry environments and mild temperatures, such as PRRSV, PEDV and coronaviruses in general [34, 47].

## Ultraviolet light irradiation

Ultraviolet exposure at a wavelength of 254 nm (UV-C) is a nonthermal process that has a germicidal effect by causing thymine-thymine and thymine-cytosine dimers in DNA and thymine-uracil dimers in RNA, which disrupts microbial reproduction [48].

Technological advancements have resulted in the development of UV-C irradiation devices that create a turbulent flow which allows for effective irradiation of opaque fluids of high viscosity. These UV devices have been widely used in the food industry for the treatment of complex opaque liquids or other substances including juices [49–53], tea [54], milk [55–57], cheese [58], wine [51], egg [59], dried seafood [60] and sliced fruits and vegetables [61–64]. UV-C irradiation technology has also been used for the inactivation of several viruses in human plasma products [65–71].

### UV irradiation effect on bacteria survival in SDP

The effectiveness of different doses of UV-C irradiation of liquid porcine or bovine plasma on survival of several bacteria of interest in farm animals including *Salmonella typhimurium (S. typhimurium)*, *Salmonella choleraesuis (S. choleraesuis), Enterococcus faecium (E. faecium),* and *Escherichia coli (E. coli K88 and K99*) has been evaluated [30, 72].

For all bacteria tested, the 4D reduction value (UV irradiation dose at which a specific microorganism reduces its viability by 4 Log_10_) was achieved around 3000 J/L, which is the dose typically applied under commercial manufacturing conditions. All bacteria showed non-linear inactivation kinetics, having special importance for *S. typhimurium* and *E. coli* K88 and K99, in which tails appeared in their inactivation kinetics curves. The appearance of non-linear kinetic tail inactivation of *S. typhimurium* excludes the potential use of *E. faecium* as its surrogate. Tails appeared in inactivation kinetics when, despite increasing the dose of UV-C, the reduction in the population slows down and is not proportional to the increase in UV-C irradiation [73].

### UV irradiation inactivation of enveloped and non-enveloped viruses in SDP

The effect of UV-C on different viruses of interest in the swine industry was also determined [74]. Viruses selected for testing was based on their different physicochemical characteristics, types of genome (DNA or RNA), genome lengths (long and short genomes), presence or absence of envelope and resistance to other inactivation processes. Also, viruses belonging to the same family and genus were selected to determine if they would have similar behaviors under UV-C irradiation. As outlined by the WHO (2004) guidelines [46], it is always prudent to test the inactivation process with the virus of interest, choosing the strain with the greatest known resistance. However, it is also important to test viruses with different physicochemical characteristics to obtain information about the robustness of the inactivation process [46].

Enveloped viruses selected for the studies included PRV, PRRSV, PEDV, *Bovine viral diarrhea virus* (BVDV), *Swine influenza virus* (SIV) and *Classical swine fever virus* (CSFV) and *Porcine parvovirus* (PPV), SVDV, PCV-2 and *Senecavirus A* (SVA) were chosen as non-enveloped viruses. All viruses were inoculated in liquid plasma and subjected to different UV-C doses. The inactivation curve for each virus was constructed by titration of the samples in their respective target cell at each UV-C dose. In general, results showed that enveloped viruses have a higher sensitivity to UV-C than non-enveloped ones, because the 4D reduction value was less than 2000 J/L for all enveloped viruses (Table 3). Furthermore, UV-C irradiation of ASFV (strain Badajoz 71 adapted to Vero cells) inoculated plasma at 3000 J/L was apparently able to reduce infectivity around 4 log_10_ TCID_50_/mL [75].

**Table 3:** Log reduction of viral titers expressed as Log 10 TCID_50_ at UV-C doses of 3000 J/L in liquid plasma (references [74, 75])

Virus	Log 10 TCID_**50**_ reduction at 3000 J/L
**PRV**	4.5
**PRRSV**	> 4.0
**PEDV**	> 4.1
**BVDV†**	> 4.2
**SIV**	> 5.1
**CSFV**	> 4.1
**ASFV**	~ 4.0
**SVDV**	2.6
**PCV-2**	1.8
**PPV**	> 4.0
**SVA**	3.7

Within the group of enveloped viruses, pestiviruses (CSFV and BVDV) had similar UV-C inactivation indicating that viruses belonging to the same genus could be used as a surrogate organism.

Regarding non-enveloped viruses, PPV and SVA had 4D reduction values very close to 3000 J/L and SVDV had a slightly higher 4D value. In the case of PCV-2, the 4D value could not be calculated because less than 4 log_10_ TCID_50_/mL was measured in the inoculated plasma before UV-C treatment. Even so, PCV-2 had higher resistance to UV-C treatment because inactivation was only 2 and 3 log_10_ TCID_50_/mL at 3000 and 9000 J/L, respectively. These results are in agreement with the available literature, confirming the well-known high resistance of PCV-2 to inactivation treatments [76].

The overall results of the use of UV-C irradiation on viruses demonstrated that it is a useful technology to significantly reduce the viral and bacterial load in plasma. Considering that UV-C can be included as a biosafety step before the spray-drying process, the reduction factor achieved by UV-C would be additive to that obtained by spray-drying, which has been demonstrated for some of these viruses as previously discussed (Table 4).

**Table 4:** Combined inactivation steps in the manufacturing process of SDP. Inactivation expressed as colony-forming unit (cfu) per g in case of bacteria and Log_10_ TCID_50_ for viruses

Bacteria /Virus (enveloped or non-enveloped)	Spray-Drying	Reference	UV-C	Reference	Storage at 20 °C for 14 d	Reference	Combined Theoretical Inactivation
*E. coli* K88	7.3	[30]	4.3	[30]			11.6
*E. coli* K99	7.7	[30]	4	[30]			11.7
*Salmonella typhimurium*	5.4	[29]	3.6	[72]	> 4.2	[29]	> 13.2
*Salmonella choleraesuis*	5.3	[29]	5.6	[72]	> 4.8	[29]	> 15.7
PRV (enveloped)	> 5.3	[31]	4.5	[74]			> 9.8
PRRSV (enveloped)	> 4.0	[31]	> 4.0	[74]	> 4.0	[47]	> 12.0
PEDV (enveloped)	> 5.2	[34]	> 4.1	[74]	> 3.5	[34]	> 12.8
ASFV (enveloped)	~ 4.0	[35]	~ 4.0	[75]			~ 8.0
SVDV (non-enveloped)	> 6.0	[32]	2.6	[74]			> 8.6

Blank cell means that the inactivation associated with that step has not been determined

Validation of the UV irradiation effect in an animal model.

To validate the effectiveness of the plasma UV-C irradiation measured by means of the viral load reduction in cell culture, a bioassay was done with different groups of pigs injected intraperitoneally with UV-C irradiated commercial liquid plasma at 0 J/L (untreated plasma), 3000 J/L, and at 9000 J/L [77]. The results of the bioassay showed that none of the pigs in the groups that received liquid plasma irradiated by UV-C at either dose became infected or seroconverted against the different virus genomes that were detected in the initial plasma (PCV-2, PRRSV (European strains), SIV, PPV, HEV, Rotavirus A (RVA); thus, confirming the efficacy of UV-C demonstrated in vitro in a previous study [74]. Detection of a viral genome in the untreated liquid plasma does not imply infectivity by a given virus. The swine bioassay was a very sensitive test to ascertain the infectiousness of the detected genome of these viruses in the plasma used in these experiments.

## Discussion

The manufacturing process of SDP involves several safety features including veterinary inspection at the abattoir, neutralizing antibodies present in pooled plasma, the spray-drying process and post-processing storage. Veterinary inspection is crucial to ensure that blood from only healthy animals slaughtered for human consumption is the exclusive source of raw material to be used for the manufacturing of blood products. Therefore, it is vital to understand inherent safety features, such as the actions of neutralizing antibodies on pathogen load, and to validate the biosafety steps that are part of the manufacturing process of SDP which are directly implemented to inactivate and/or eliminate pathogens.

The action of neutralizing antibodies to endemic pathogens in the population has been demonstrated in various studies [12–14] to be an inherent safety step that may contribute to the safety of blood products. The spray-drying conditions used during the manufacturing of SDP represent the most critical step that contributes to the inactivation of different pathogens [28, 31–34]. Also, certain storage conditions (such as room temperature > 20 °C for 14 days) have been demonstrated as a safety treatment for some enveloped viruses and bacteria [34, 47]. All of these biosafety steps contribute to the global safety of SDP, as demonstrated in several in vivo studies [36–39, 43, 45, 78].

Since SDP is produced from plasma with high protein concentration and biological activity, it is important that these proteins be preserved during processing. UV-C irradiation was profiled as a good candidate to be implemented in the manufacturing process as a new redundant biosafety step because of its limited impact on biologically active proteins that benefit animal health. Technological advancements have resulted in the development of UV-C irradiation devices based on turbulent flow, which enables the irradiation of opaque fluids with high viscosity efficiently. In addition, these UV devices have been widely used in the food industry for the treatment of other complex opaque liquids like milk or fruit juices.

Despite that the implementation of UV-C and spray-drying as inactivation processes has shown its effectiveness in reducing bacterial and viral loads in plasma, the presence of some of these pathogen genomes (especially viruses) detected in SDP by real time PCR (qRT-PCR) generates doubts about its potential infectivity. The biosafety steps to produce SDP inactivate several pathogens, but the pathogen genome is not eliminated from the final product and the genetic material can be detected by PCR techniques. However, PCR techniques are not able to differentiate between infective and non-infective viral particles [46]. In vivo bioassays or feeding studies are still considered the most accurate method of distinguishing if viral particles in SDP are infective or not, and as previously discussed SDP has shown to be non-infective in feeding studies, even though viral genome was present in SDP.

The UV-C treatment for the tested pathogenic bacteria and viruses showed Log_10_ reduction values very close or superior to the 4D value using industrial manufacturing conditions (3000 J/L). Recommendations of WHO (2004), which were developed for the evaluation of inactivation/removal of viruses in human plasma derivatives, indicates that the use of two different inactivation methods with different mechanisms of action represent redundant biosafety steps. Therefore, the combination of UV-C irradiation of the liquid followed by spray-drying at 80 °C throughout its substance must be considered redundant biosafety steps for production of SDP. The Log_10_ reduction factors of each of the steps should be considered cumulative within the manufacturing process, thus increasing the overall inactivation capacity of the system (Table 4).

## Conclusion

The manufacturing process involves several safety features that mitigate any biological risk for SDP use as an ingredient in feed for pigs. The collection of blood from healthy animals and the spray-drying process are two of the safety steps that have been proven to inactivate numerous pathogens of interest for the swine industry. Furthermore, the presence of neutralizing antibodies may be considered as an additional inherent safety step for pathogens that are able to produce neutralizing antibodies. Also the post-processing storage of SDP at 20 °C for 14 days has proven effective for certain enveloped viruses like coronaviruses and PRRSV, while prolonged storage before release for sale provides more time for authorities to react to an outbreak of a foreign animal disease in the manufacturing region.

Overall results obtained with UV-C irradiation were effective for inactivating a diversity of bacteria and viruses spiked and naturally present in commercially collected liquid animal plasma. Since the UV-C mechanism of inactivation targets nucleic acids and is different than the thermal inactivation of the spray-drying process, UV-C can be considered an independent biosafety step in the manufacturing process of SDP. UV-C as a safety step complies with the WHO recommendations for the design of redundant biosecurity steps in the manufacturing process of human blood products for medical use. Furthermore, UV-C technology can be incorporated into the manufacturing process, and in fact it is already used in some spray-drying industrial plants. In conclusion, UV-C irradiation of liquid plasma is a suitable additional inactivation step for the industrial production process of SDP that further supports the biosafety of SDP use in animal feed.

## Data Availability

The datasets used and/or analysed during the current study are available from the corresponding author on reasonable request.

## Abbreviations

ASFV: African swine fever virus
CIP: Clean in place
DNA: Deoxyribonucleic acid
EAPA: European animal protein association
GMP: Good manufacturing practices
HEV: Hepatitis E virus
NASDBPP: North American spray dried blood and plasma producers.
PCV2: Porcine circovirus type 2
PEDV: Porcine epidemic diarrhea virus
PRRSV: Porcine reproductive and respiratory syndrome virus
PRV: Pseudorabies virus
qRT-PCR: Quantitative real time- polymerase chain reaction
RBC: Red blood cells
RNA: Ribonucleic acid
RVA: Rotavirus A
SDBP: Spray dried bovine plasma
SDP: Spray dried plasma
SDPP: Spray dried porcine plasma
SVDV: Swine vesicular disease virus
TCID_50_: 50% Tissue culture infective dose (measure of infectious virus titer)
